# Preliminary results of a triple peptide escalating dose vaccination phase I/II clinical trial as consolidation treatment in women affected by ovarian cancer

**DOI:** 10.1186/2051-1426-1-S1-P227

**Published:** 2013-11-07

**Authors:** Chiara Napoletano, Valeria Visconti, Morena Antonilli, Ilaria G Zizzari, Hassan Rahimi, Federico Battisti, Salvatore Caponnetto, Giacomo Barchiesi, Aurelia Rughetti, Filippo Bellati, Pierluigi Benedetti-Panici, Marianna Nuti

**Affiliations:** 1 Department of Experimental Medicine, Sapienza University, Rome, Italy; 2 Department of Gynaecology and Obstetric, Sapienza University, Rome, Italy

## 

Ovarian cancer remains one of the most lethal malignancies in developed countries and recently no improvement in disease free survival (DFS) and overall survival (OS) has been numbered for conventional therapies. Immunotherapy could be employed as consolidation treatment after standard therapies in patients in which disease and immune suppression is minimal. Furthermore, epitope spread and new adjutants have been found to significantly increase the efficacy of novel immunotherapic strategies.This Phase I/II study aimed to improve vaccine potency and enhance immune response of two mucins (MUC-1 and CEA) and Erb-B2 tumour associated antigens (TAAs), with the co-administration of a new adjuvant combination: Montanide ISA51 plus GM-CSF. Keyhole Limpet Hemocyanin (KLH) was adopted as immunological tracker.Ten disease-free HLA-A2 aplotype high-risk serous advanced stage ovarian cancer patients underwent a two-groups safety clinical trial vaccination protocol at “Sapienza” University of Rome from 2007 to 2010. CEA, Erb-B2 and MUC1 expression rates on primary tumours were 40%, 30% and 100%, respectively. Group A (8 patients) received 100μg of each peptide per dose accordingly to the immunohistochemically antigen expression, whereas GroupB (2 patients) was vaccinated with all the peptide regardless TAAs expression at the dose of 500μg each. Vaccination schedule included 6 consecutive peptide vaccine administrations and a recall dose at 3 months from the sixth dose. Results revealed the vaccine to be safe and feasible in ovarian cancer patients. Side effects accounts for ECOG scale 1-2 levels signs (itch, erythema and tumescence at injection site) and symptoms (fever, fatigue and malaise), for both the dose-administered. Good compliance was found to vaccine schedule in all patients enrolled. Secondary endpoints results revealed a 5 years 50% (3/6 patients) DFS and a 83% (5/6 patients) OS in FIGO stages IIIc-IV ovarian cancer patients (60%; 6/10 patients). Moreover, immunological and clinical correlation analysis revealed a significant increased IFNγ CD8+ specific T-cells production along with vaccination steps (χ2=6.67; p<0.009) in the subgroup of patients who did not recur vs. controls (mean follow up: 845.1 days; range 55-1523). The highest number of vaccine induced specific CD8+ cells were found at the end of the 6 doses although high levels of T-cells could be also re-induced by the recall boost. A significantly higher specific immune response was observed in the group B.

